# Association of Social Jetlag with the Dietary Quality Among Korean Workers: Findings from a Nationwide Survey

**DOI:** 10.3390/nu16234091

**Published:** 2024-11-27

**Authors:** Seong-Uk Baek, Jin-Ha Yoon

**Affiliations:** 1 Graduate School, Yonsei University College of Medicine, Seoul 03722, Republic of Korea; 2 Department of Preventive Medicine, Yonsei University College of Medicine, Seoul 03722, Republic of Korea; 3 The Institute for Occupational Health, Yonsei University College of Medicine, Seoul 03722, Republic of Korea

**Keywords:** circadian misalignment, diet, eating habit, sleep health, Korea Healthy Eating Index

## Abstract

**Background/Objectives:** Social jetlag, which refers to the misalignment between biological rhythms and social schedule, is linked to an increased risk of metabolic diseases. This cross-sectional study investigated the relationship between social jetlag and workers’ dietary quality. **Methods**: This secondary data analysis included a sample of workers from the Korea National Health and Nutrition Examination Survey (*n* = 11,430). Social jetlag was determined by calculating the difference in the sleep midpoint between free days and workdays, based on sleep onset and offset times. The Korean Health Eating Index (KHEI) was calculated based on 24-h recalls, with higher scores indicating superior dietary qualities (range: 0–100). Poor dietary quality was defined as a KHEI below the lowest quartile (<51.6). Linear or logistic regressions were utilized to estimate *β* or odds ratio (OR), respectively. **Results:** Among study participants, 12.1% of workers experienced ≥120 min of social jetlag. ≥120 min of social jetlag was associated with a reduced KHEI score compared with 0–59 min (*β*: −1.23, 95% confidence interval [CI]: −2.16, −0.30). Those with ≥120 min of social jetlag were more likely to have poor dietary quality than those with 0–59 min (OR: 1.27, 95% CI: 1.08, 1.50). **Conclusions**: Workers experiencing ≥120 min of social jetlag had poorer dietary quality compared with workers with 0–59 min of social jetlag. Therefore, this study suggests that policy efforts are needed to reduce social jetlag among workers in Korea.

## 1. Introduction

Social jetlag, which refers to the misalignment between biological clock and social schedule, has garnered increasing academic interest in recent years [1,2]. Social jetlag often arises when workers are expected to align their daily routines with social schedules during weekdays, predominantly affecting workers who wake up earlier than their circadian rhythm prefers [3]. According to previous studies, approximately 70% of workers or students had at least 1 h of social jetlag, which was quantified as the disparity between the midpoint of sleep initiation and termination on workdays and non-workdays [3,4]. Although its prevalence is lower in Korean workers than in Western populations, certain demographics, including younger workers, those with high educational and income levels, and white-collar workers, experience high social jetlag, according to previous studies [5,6,7].

In the existing literature, social jetlag has been associated with various cardiometabolic risk factors, including overweight/obesity, components of metabolic syndrome, and diabetes mellitus [8,9,10]. The link between social jetlag and metabolic abnormalities is assumed to be mediated by complex mechanisms, including disruption of the hypothalamic–pituitary–adrenal axis and behavioral changes [8,10]. Previous research demonstrated that social jetlag was linked to undesirable dietary behaviors [11], a negative correlation between social jetlag and the intake of vegetables, fruits, or grains [12,13,14], and a positive correlation with the intake of sugar, sweets, and fast food/junk food [13,15]. However, current evidence pertaining to the association between social jetlag and dietary intake remains mixed and inconclusive, as indicated by a recent systematic review [11].

Compared to the evaluation of individual food components or nutrients, dietary quality assessment adopts a more holistic approach, considering the interactive and synergistic effects among nutritional components. Previous studies have demonstrated a negative association between social jetlag and various dietary quality indices, including the Mediterranean Diet Quality Index [16], Baltic Sea Diet Score [12], Japanese Food Guide Score [17], and an empirically driven scoring system from the United Kingdom (UK) population [18]. In contrast, other studies have found no clear association between social jetlag and dietary quality index [19,20]. However, the existing literature has several limitations. For example, most studies have focused on Western populations and the dietary practices of the corresponding regions, resulting in a scarcity of research in the Asian context. Although two Japanese studies investigated the association of social jetlag with dietary quality, the results produced mixed findings [17,20]. To the best of our knowledge, the relationship between social jetlag and dietary quality among the Korean population has not yet been examined. Another limitation is that most studies have relied on small sample sizes, highlighting the need for research that includes nationally representative samples.

The Korea Healthy Eating Index (KHEI) is a validated measure of dietary quality for the Korean population. Prior research has consistently indicated a negative association between the KHEI and metabolic disorders and obesity [21,22,23]. It has been integrated into the Korea National Health and Nutrition Examination Survey (KNHANES), an annual nationwide health survey. The objective of our study was to examine the association between social jetlag and dietary quality, as assessed using the KHEI, in the general working population in Korea.

## 2. Methods

### 2.1. Study Population

The KNHANES, an annual cross-sectional survey administered by the Korean government, served as the source for the study sample [24]. The study utilizes a multi-stage cluster sampling method. Administrative regions in Korea serve as the primary sampling units, while households within these districts are the secondary sampling units. Consequently, the KNHANES selects a nationally representative sample of the Korean population [24]. The response rate to the KNHANES nutrition survey was approximately 80.0% in each survey year [25]. For the selected households, a survey was conducted by trained interviewers using household visits. To further mitigate the bias from non-responses and improve the generalizability of the sample, the KNHANES calculated a survey weight for each survey participant.

**Figure 1:**
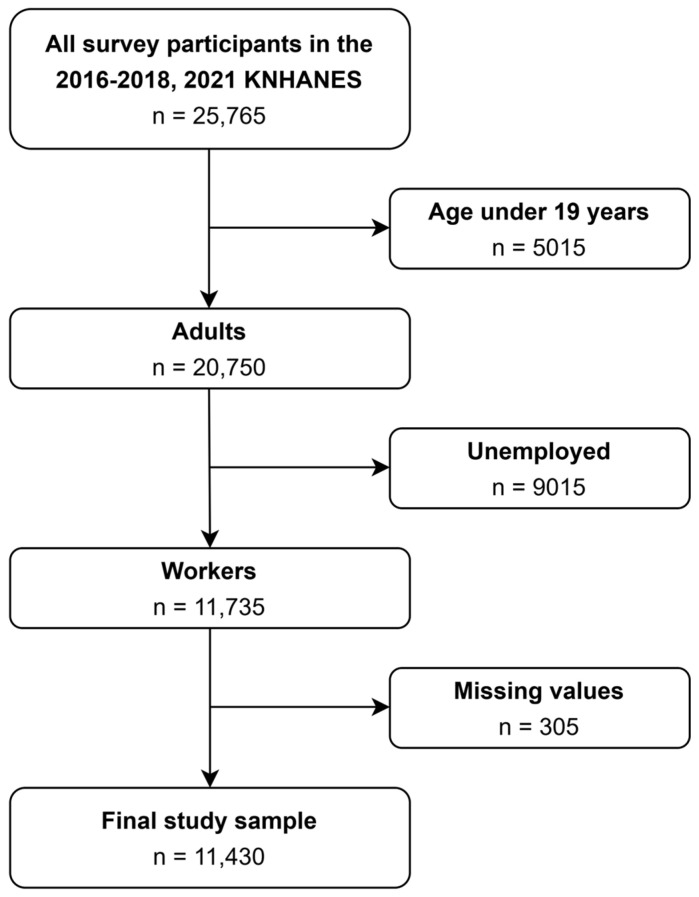
Diagram of the sample selection procedure.

Figure 1 shows the sample selection process. Our secondary data analysis included participants from the 2016, 2017, 2018, and 2021 KNHANES, during which sleeping times were collected for both workdays and free days. Initially, 25,765 participants were included. We then limited our study sample using the following criteria: (1) adult (age >18 years), (2) workers, (3) workers without missing values. Finally, 11,430 workers were analyzed. Data are obtainable at https://knhanes.kdca.go.kr/ (accessed on 12 December 2023). The Institutional Review Board of Severance Hospital approved the protocol of our study (4–2023–0959). 

### 2.2. Variables

#### 2.2.1. Social Jetlag (Exposure)

Social jetlag was quantified as the disparity between the midpoint of sleep onset and offset on workdays and non-workdays, as described in the following equation [3]:

Social jetlag=MSF−MSW

In the above equation, the MSF indicates the midpoint of sleep onset and offset on free days, which was assessed by the following question: “Typically, what time do you go to bed and wake up on free days (or weekends)?” The MSW indicates the midpoint of sleep onset and offset on workdays, which was assessed by the following question: “Typically, what time do you go to bed and wake up on workdays (or weekdays)?” The responses to each question were gathered in hours and minutes. Following prior research investigating the association of social jetlag with health [5,26], we categorized social jetlag as “<0 min”, “0–59 min”, “60–119 min”, and “≥120 min”, in which “0–59 min” was considered the reference group. Individuals with negative values of social jetlag (<0 min) had earlier sleep schedules on free days compared to workdays, primarily observed among those with an early chronotype or older adults [1,2,27]. Workers with 0–59 min of social jetlag were considered the reference group, as this range is generally accepted as showing normal variations in weekday–weekend sleep schedules in the previous literature [5,26].

#### 2.2.2. Dietary Quality Assessment (Outcome)

The dietary quality was assessed using the KHEI. Developed as an evidence-based dietary quality assessment tool for Korean adults, the KHEI is based on Korean dietary guidelines [28]. The KHEI consists of 14 items and 3 subcomponents, where 8 items assess the appropriate intake of recommended foods, including vegetables, fruits, and mixed grains (adequacy component); 3 items assess the proper intake of sweets and beverages, sodium, and saturated fatty acid (moderation component); and 3 items assess the balanced intake of fat, carbohydrate, and total energy (energy balance component). For the adequacy component, the daily serving of each food was evaluated for each respondent to calculate scores for individual items, with higher scores designed to reflect greater intake. For the moderation component, the intake amounts of saturated fatty acids, sodium, sweets, beverages, and alcoholic drinks were calculated, with higher scores intended for lower energy intake. Regarding the energy balance component, consuming a predetermined percentage of carbohydrates and fats relative to total energy intake, and having proper energy intake based on demographic characteristics, were designed to yield higher scores. The total KHEI score ranges from 0 to 100, higher scores indicating better dietary qualities. For the regression analysis, the KHEI score was included as the continuous dependent variable. Additionally, we defined poor dietary quality as a KHEI < 51.6, which corresponds to the lowest quartile in the study sample, in accordance with the methodology employed in previous studies [29,30]. The detailed scoring system of the KHEI has been elaborated by Yun et al. [28]. The survey was developed by the Korean government, and the KHEI was collected using the 24-h recall method with standardized protocols applied to minimize biases. Detailed information can be found in previous literature [25,31,32,33].

#### 2.2.3. Covariates

The selection of the covariates was informed from the prior research on the association between social jetlag and dietary quality [12,16,17,18,34]. Sex (male, female) was adjusted for. Age was grouped into <40, 40–49, 50–59, and ≥60. Education level was grouped into middle school or below, high school, and college or above. Income level was grouped into four categories (lowest, low, high, highest) using the quartile values of total monthly wage for each year. Marital status was grouped into married, unmarried, or other (divorced, separated, or widowed). Job was grouped into blue-collar jobs, service or sales workers, or white-collar. Working hours were grouped into ≤52 h per week and >52 h per week [35]. Shift work was grouped into “yes” or “no”. Chronic conditions were grouped into yes or no based on whether the respondent had at least one of the following diseases: diabetes, dyslipidemia, or hypertension. Current smoking status was adjusted (yes or no). Physical activity was grouped into yes or no, depending on whether respondents participated in ≥150 min of physical activity per week, based on the current physical activity guidelines [36]. Obesity statistics were determined as a body mass index of ≥25 kg/m^2^. Perceived stress was assessed using the question, “To what extent do you experience stress in your daily life?” Participants who responded with “very much” or “a lot” were classified as experiencing perceived stress (“yes”), while those who indicated “a little” or “hardly any” were classified as not experiencing perceived stress (“no”). Finally, the survey year was controlled for regression models.

### 2.3. Aanlysis Plan

We first investigated the distribution of the KHEI within the sample using the histogram and Q-Q plot. Subsequently, linear regressions were utilized to estimate *β* and 95% confidence interval (CI) for the association between social jetlag and the KHEI. We then estimated odds ratio (OR) for the association between social jetlag and poor dietary quality (KHEI < 51.6) using logistic regression models. Finally, we examined the nonlinear association of continuous social jetlag values with the KHEI and the probability of poor dietary quality using the restricted cubic spline function. R software (version 4.4.1) was utilized for analyses. The sampling weights of the individuals were incorporated into both descriptive and regression analyses. The “survey” package in R software was used.

## 3. Results

The study sample comprises 5704 (58.3%) men and 5726 (41.7%) women (Table 1). Among the study sample, 5.0% had social jetlag of <0 min, 58.0% had 0–59 min, 24.9% had 60–119 min, and 12.1% had ≥120 min. Workers experiencing ≥120 min of social jetlag were more likely to be young, have a higher education level, have white-collar occupations, and be unmarried compared to workers with 0–59 min of social jetlag. This suggests a correlation between greater social jetlag and characteristics commonly associated with younger individuals and those of higher socioeconomic status. Such individuals may experience more irregular schedules and social commitments compared to those with lower levels of social jetlag.

Participants with ≥120 min of social jetlag had lower mean KHEI scores than those with 0–59 min of social jetlag (56.3 vs. 62.4; Table 2). Individuals experiencing ≥120 min of social jetlag exhibited lower scores on items related to the intake of mixed grains, fruits, and vegetables than those with 0–59 min of social jetlag. Furthermore, participants with ≥120 min of social jetlag exhibited lower scores in items related to moderated intake of saturated fatty acids, sodium, and sweets/beverages/alcohol than those with 0–59 min of social jetlag. These results suggest a non-linear relationship between social jetlag and KHEI. The highest KHEI scores were observed in the 0–59 min category, declining as values shifted towards the negative range or exceeded 59 min. Furthermore, the lowest average KHEI scores were observed among workers experiencing ≥120 min of social jetlag. The results indicate that individuals with a social jetlag of 120 min or more exhibited the lowest dietary quality across all three domains of the KHEI, including adequacy, moderation, and energy balance components, compared to the other three categories of social jetlag. The distribution of KHEI in the sample is shown in Figures S1 and S2.

The associations among social jetlag, the KHEI, and poor dietary quality based on regression models are presented in Table 3. In Model 3, the *β* (95% CI) of the association of social jetlag with the KHEI was −1.03 (−2.27, 0.21) for <0 min, −0.54 (−1.19, 0.12) for 60–119 min, and −1.23 (−2.16, −0.30) for ≥120 min in comparison to 0–59 min. Additionally, the OR (95% CI) of the association of social jetlag with poor dietary quality was 1.24 (0.96, 1.60) for <0 min, 1.12 (0.97, 1.28) for 60–119 min, and 1.27 (1.08, 1.50) for ≥120 min in comparison to 0–59 min. Overall, these results indicate that greater social jetlag is associated with lower dietary quality as measured by the KHEI. High social jetlag (≥120 min) is particularly concerning, as it consistently shows negative associations with dietary quality across various models, even after adjusting for potential confounders. Additionally, the regression models also suggest a non-linear association between social jetlag and dietary quality, as shown in Table 2.

**Figure 2:**
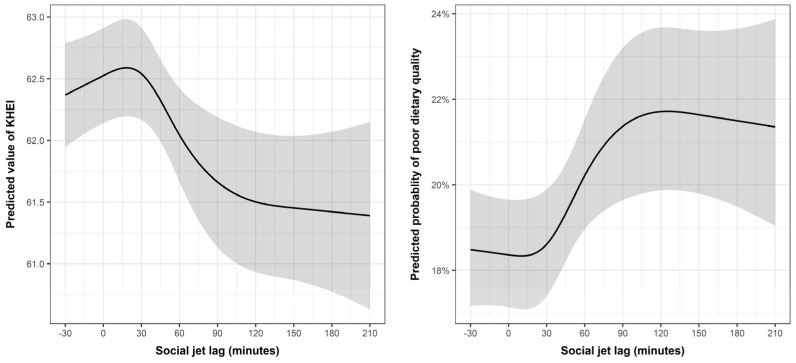
Nonlinear association of social jetlag with the KHEI score and poor dietary quality among workers (*n* = 11,430). Notes: Grey area indicates the confidence interval. Models adjusted for sex, age, education, income, marital status, job, working hours, shiftwork, chronic conditions, smoking status, physical activity, obesity, perceived stress, and survey year. Poor dietary quality was defined as KHEI ≤ 51.6, which corresponds to the lowest quartile of the KHEI score in the study sample. (KHEI, Korean Healthy Eating Index).

Figure 2 shows the nonlinear association of minutes of social jetlag with the KHEI score and probability of poor dietary quality. The results show that the predicted value of the KHEI reached its peak at 0–30 min of social jetlag and subsequently decreased with further increments in social jetlag beyond this threshold.

Table 4 presents the results of each nonlinear analysis, presenting the highest and lowest points observed in Figure 2. It shows that the highest dietary quality was associated with social jetlag of 10–18 min, while the poorest dietary quality was linked to 125–210 min of social jetlag. This finding aligns with the regression analysis presented in Table 3. The results indicate that a social jetlag of 120 min or more is correlated with poor dietary quality.

## 4. Discussion

Our study revealed that ≥120 min of social jetlag was negatively associated with the KHEI in a nationwide sample of Korean workers compared to 0–59 min of social jetlag. Additionally, one of the novel findings of this study was to identify a nonlinear association between social jetlag and dietary quality, with the highest dietary quality observed at 0–59 min of social jetlag. Specifically, both an increase in social jetlag values beyond 59 min and a decrease in social jetlag values below 0 min were correlated with a decline in KHEI scores. To the best of our knowledge, this study is the first to examine the association between social jetlag and dietary quality among Korean workers, providing a meaningful contribution to the literature.

Our descriptive analyses showed that a high level of social jetlag was particularly noticeable among unmarried, young-aged individuals. This is consistent with the findings from a previous study that young-aged workers are more likely to have high levels of social jetlag [1]. This phenomenon may be attributed to the fact that young individuals are more likely to have a late chronotype and are exposed to artificial light from smartphones or computers more frequently [1,4]. Therefore, there is a need for targeted policy efforts to mitigate social jetlag among young-aged workers. In particular, our analysis showed that workers with social jetlag exceeding 120 min were linked to skipping meals, consuming fewer fruits and vegetables, and having unrestricted intake of saturated fatty acids. This highlights the potential necessity for workplace programs aimed at mitigating social jetlag of workers [37]. For example, flexible work hours could help align workers’ sleep schedules with their natural circadian rhythms, thereby reducing social jetlag. Additionally, reducing excessive exposure to blue light during work hours may help mitigate social jetlag of workers [38].

The results of this study accord with prior studies, indicating that social jetlag is linked to poor dietary quality. For instance, ≥120 min of social jetlag was correlated with lower dietary quality index in a Japanese working population compared to <60 min of social jetlag [17]. Similarly, social jetlag has been related to reduced adherence to the Mediterranean Diet among young Spanish adults [16] and poor dietary quality in Finnish adults [12]. A recent study also showed that ≥1.5 h of social jetlag was related to unhealthful plant-based Diet Index in a healthy UK population [39]. However, there are several studies that contradict the results of this study. For example, studies from Turkey and Japan observed no significant difference in the dietary quality between university students with or without social jetlag [19,20]. The variance in study findings could stem from differences in sample characteristics and size, as well as regional disparities in dietary practices. Dietary practices and cultures vary considerably across regions, leading to the use of region-specific dietary guidelines for assessing dietary quality in relation to social jetlag. To the best of our knowledge, this study is the first to demonstrate an association of social jetlag with poor dietary quality among Korean adults. Notably, in contrast to previous studies, our study benefits from the analysis of a nationally representative sample, enhancing the generalizability of the findings.

Several mechanisms may explain the relationship between social jetlag and poor dietary quality. First, circadian misalignment can contribute to alterations in appetite and hunger. Previous studies have suggested that sleep deprivation, often stemming from social jetlag, is linked to an increase in ghrelin levels, leading to heightened appetite and hunger [40,41]. Additionally, experiencing social jetlag and sleep deprivation may increase stress in workers, resulting in uninhibited eating and greater need for energy to endure prolonged wakefulness during workdays [13,42]. However, the potential influence of poor dietary quality on social jetlag should be considered. For instance, a previous cohort study also indicated that dietary pattern characterized by high consumption of “Western” processed foods resulted in higher social jetlag at follow-up [34]. Therefore, future longitudinal studies exploring the reciprocal relationship between social jetlag and dietary quality are warranted.

This study had several limitations. First, the causal effects of social jetlag could not be determined. We could not consider the effect of certain potential confounding factors, including personality traits or job strain, because of lack of information. Additionally, lifestyle factors, socioeconomic status, and mental health conditions may influence both social jetlag and dietary quality; however, we could not fully address these factors in our analysis. Second, the cross-sectional design prevented us from examining the temporal relationship between social jetlag and dietary quality. Specifically, the possibility of reverse causation, in which poor dietary quality could induce social jetlag among workers, should be considered. For example, previous research suggests that a dietary pattern characterized by high consumption of meat and starchy foods is associated with higher social jetlag among Mexican adolescents [34]. Therefore, the estimates in this study should be interpreted as associations rather than causal effects of social jetlag on dietary quality, within a bidirectional framework. Future longitudinal studies are needed to clarify the temporal relationship between social jetlag and changes in dietary quality. Third, although the KHEI is a validated tool for evaluating the dietary quality of Korean adults, the scoring system is based on 24-h recalls, which may lead to potential measurement errors, including recall bias. Furthermore, we acknowledge that relying on 24-h recalls may not capture the usual dietary quality and variations in dietary intake between workdays and free days. Therefore, future studies should incorporate more advanced dietary assessment methods, such as multiple recalls or a food frequency questionnaire, to obtain a more reliable measurement of long-term dietary quality. Fourth, to address potential interviewer bias, dietary assessments were carried out by trained dieticians following a standardized protocol and underwent annual quality control checks, as detailed in the methods section. However, we acknowledge the possibility of interviewer bias, as data collection may vary depending on the dieticians involved. Fifth, the evaluation of social jetlag relied on self-reporting. Consequently, future studies could benefit from incorporating objective measurements. Fifth, one of the limitations of this study is its focus on Korean workers, which may restrict the generalizability of the findings to other populations. Given the significant cultural and regional disparities in dietary practices, these findings should be interpreted with caution when applied to other contexts.

Nonetheless, this study has some strengths. First, it was based on a nationwide sample of Korean workers, which enhances the generalizability of our findings. Moreover, this is the first study to report a meaningful correlation between social jetlag and dietary quality in a Korean population. Considering that the link between social jetlag and dietary intake has predominantly been explored in specific regions, such as certain Western countries, we believe that this study makes a meaningful contribution.

## 5. Conclusions

In our study, social jetlag ≥120 min was linked to poorer dietary quality measured by the KHEI compared with social jetlag of 0–59 min in workers. Although future longitudinal studies are needed to elucidate causal relationships, our findings suggest a potential association between social jetlag and poor dietary quality. Therefore, policy interventions are required to mitigate high levels of social jetlag among workers.

## Figures and Tables

**Table 1:** Characteristics of the study sample by social jetlag.

	Overall	Social Jetlag (min)			
		<0	0–59	60–119	≥120
	*n* = 11,430	*n* = 542	*n* = 7093	*n* = 2645	*n* = 1150
Sex					
Male	5704 (58.3)	281 (60.3)	3635 (59.4)	1205 (55.0)	583 (58.9)
Female	5726 (41.7)	261 (39.7)	3458 (40.6)	1440 (45.0)	567 (41.1)
Age					
<40	3258 (36.2)	144 (35.2)	1413 (26.6)	1023 (44.4)	678 (66.2)
40–49	2627 (24.8)	110 (22.2)	1447 (23.6)	829 (31.1)	241 (19.0)
50–59	2592 (23.1)	133 (24.7)	1825 (27.8)	485 (17.7)	149 (11.3)
≥60	2953 (15.8)	155 (17.9)	2408 (22.1)	308 (6.8)	82 (3.6)
Education level					
Middle school or below	2724 (16.4)	164 (22.5)	2146 (21.6)	305 (8.2)	109 (5.9)
High school	3742 (34.7)	196 (40.1)	2226 (33.4)	868 (34.2)	452 (40.1)
College or above	4964 (48.9)	182 (37.4)	2721 (45.0)	1472 (57.7)	589 (54.0)
Income level					
Lowest	2347 (20.4)	115 (20.8)	1501 (21.2)	487 (18.9)	244 (19.8)
Low	2993 (25.9)	150 (25.8)	1873 (25.9)	668 (25.4)	302 (27.3)
High	3059 (26.7)	160 (31.0)	1872 (26.3)	726 (27.3)	301 (26.2)
Highest	3031 (26.9)	117 (22.4)	1847 (26.7)	764 (28.4)	303 (26.8)
Marital status					
Married	8219 (67.9)	367 (64.6)	5455 (74.7)	1886 (67.1)	511 (38.7)
Unmarried	1990 (23.8)	97 (24.5)	745 (15.2)	594 (28.1)	554 (56.1)
Others	1,221 (8.2)	78 (10.9)	893 (10.1)	165 (4.8)	85 (5.2)
Job					
White collar	4597 (44.2)	158 (31.7)	2470 (39.8)	1414 (54.0)	555 (50.1)
Service/sales worker	2392 (21.2)	153 (30.1)	1545 (22.5)	466 (17.1)	228 (20.1)
Blue collar	4441 (34.6)	231 (38.2)	3078 (37.7)	765 (28.9)	367 (29.8)
Working hours					
≤52 h	9456 (82.3)	399 (73.5)	5826 (81.6)	2273 (85.6)	958 (83.1)
>52 h	1974 (17.7)	143 (26.5)	1267 (18.4)	372 (14.4)	192 (16.9)
Shift work					
No	9668 (83.5)	326 (60.3)	6070 (84.2)	2345 (87.6)	927 (81.1)
Yes	1762 (16.5)	216 (39.7)	1023 (15.8)	300 (12.4)	223 (18.9)
Chronic condition					
No	7217 (68.1)	315 (65.1)	4066 (62.5)	1930 (75.0)	906 (81.6)
Yes	4213 (31.9)	227 (34.9)	3027 (37.5)	715 (25.0)	244 (18.4)
Current smoking					
No	9170 (76.3)	424 (75.1)	5801 (78.2)	2134 (76.7)	811 (67.2)
Yes	2260 (23.7)	118 (24.9)	1292 (21.8)	511 (23.3)	339 (32.8)
Physical activity					
No	6365 (52.1)	301 (51.8)	4156 (54.8)	1343 (48.5)	565 (46.7)
Yes	5065 (47.9)	241 (48.2)	2937 (45.2)	1302 (51.5)	585 (53.3)
Obesity					
No	7323 (63.3)	326 (58.8)	4467 (62.3)	1777 (65.6)	753 (65.1)
Yes	4107 (36.7)	216 (41.2)	2626 (37.7)	868 (34.4)	397 (34.9)
Percieved stress					
No	8294 (71.6)	380 (68.3)	5309 (73.8)	1878 (71.1)	727 (63.0)
Yes	3136 (28.4)	162 (31.7)	1784 (26.2)	767 (28.9)	423 (37.0)

Values are presented as *n* (weighted %).

**Table 2:** Characteristics of the Korean Healthy Eating Index by social jetlag.

	Score	Overall	Social Jetlag (min)			
			<0	0–59	60–119	≥120
	Range	*n* = 11,430	*n* = 542	*n* = 7093	*n* = 2645	*n* = 1150
Total KHEI score	0–100	60.9 ± 13.4	60.0 ± 13.6	62.4 ± 13.2	60.0 ± 13.3	56.3 ± 13.4
Adequacy component						
Total adequacy score	0–55	29.8 ± 10.5	28.7 ± 10.9	30.9 ± 10.3	29.0 ± 10.4	26.7 ± 10.2
Have breakfast	0–10	6.6 ± 4.1	6.4 ± 4.2	7.3 ± 3.9	6.0 ± 4.1	4.9 ± 4.1
Mixed grains intake	0–5	1.8 ± 2.1	1.8 ± 2.1	2.0 ± 2.1	1.6 ± 2.0	1.3 ± 1.9
Total fruits intake	0–5	1.9 ± 2.1	1.7 ± 2.0	2.1 ± 2.2	1.8 ± 2.1	1.4 ± 2.0
Fresh fruits intake	0–5	2.1 ± 2.3	1.9 ± 2.3	2.3 ± 2.4	2.0 ± 2.3	1.6 ± 2.2
Total vegetables intake	0–5	3.5 ± 1.5	3.5 ± 1.5	3.6 ± 1.4	3.4 ± 1.5	3.2 ± 1.5
Vegetables intake excluding Kimchi and pickled vegetables intake	0–5	3.2 ± 1.6	3.1 ± 1.6	3.3 ± 1.6	3.1 ± 1.6	3.0 ± 1.6
Meat, fish, eggs, and beans intake	0–10	7.4 ± 2.9	7.3 ± 3.1	7.3 ± 3.0	7.6 ± 2.9	7.5 ± 2.9
Milk and milk products intake	0–10	3.3 ± 4.4	3.0 ± 4.3	3.2 ± 4.4	3.5 ± 4.5	3.7 ± 4.5
Moderation component						
Total moderation score	0–30	21.9 ± 6.1	21.9 ± 6.1	22.3 ± 5.9	21.4 ± 6.1	20.5 ± 6.2
Percentage of energy from saturated fatty acid	0–10	7.0 ± 4.1	7.0 ± 4.1	7.3 ± 4.0	6.6 ± 4.3	6.2 ± 4.4
Sodium intake	0–10	6.5 ± 3.3	6.3 ± 3.4	6.5 ± 3.3	6.4 ± 3.3	6.3 ± 3.4
Percentage of energy from sweets, beverages, and alcoholic drinks	0–10	8.5 ± 3.0	8.6 ± 3.0	8.5 ± 3.0	8.4 ± 3.1	8.1 ± 3.4
Energy balance component						
Total energy balance score	0–15	9.3 ± 4.6	9.4 ± 4.7	9.2 ± 4.7	9.6 ± 4.5	9.1 ± 4.8
Percentage of energy intake from carbohydrate	0–5	2.7 ± 2.1	2.8 ± 2.1	2.6 ± 2.1	2.9 ± 2.1	2.7 ± 2.1
Percentage of energy intake from fat	0–5	3.5 ± 2.1	3.5 ± 2.0	3.4 ± 2.1	3.6 ± 2.0	3.5 ± 2.1
Energy intake	0–5	3.1 ± 2.2	3.1 ± 2.2	3.1 ± 2.2	3.1 ± 2.2	2.8 ± 2.3

Values are presented as weighted mean ± standard deviation. KHEI: Korean Healthy Eating Index.

**Table 3:** Association of social jetlag with the Korean Healthy Eating Index and poor dietary quality.

	Model 1	Model 2	Model 3
	*β* (95% CI)	*β* (95% CI)	*β* (95% CI)
Total KHEI score ^a^			
Social jetlag			
<0 min	−2.38 (−3.72, −1.04)	−1.56 (−2.84, −0.29)	−1.03 (−2.27, 0.21)
0–59 min	0.00 (0.00, 0.00)	0.00 (0.00, 0.00)	0.00 (0.00, 0.00)
60–119 min	−2.43 (−3.12, −1.73)	−0.35 (−1.03, 0.33)	−0.54 (−1.19, 0.12)
≥120 min	−6.09 (−7.05, −5.14)	−2.38 (−3.33, −1.43)	−1.23 (−2.16, −0.30)
	**Model 1**	**Model 2**	**Model 3**
	**OR (95% CI)**	**OR (95% CI)**	**OR (95% CI)**
Poor dietary quality ^b^			
Social jetlag			
<0 min	1.48 (1.18, 1.86)	1.35 (1.06, 1.71)	1.24 (0.96, 1.60)
0–59 min	1.00 (1.00, 1.00)	1.00 (1.00, 1.00)	1.00 (1.00, 1.00)
60–119 min	1.43 (1.26, 1.62)	1.08 (0.95, 1.24)	1.12 (0.97, 1.28)
≥120 min	2.31 (1.99, 2.68)	1.45 (1.24, 1.69)	1.27 (1.08, 1.50)

KHEI, Korean Healthy Eating Index; OR, odds ratio; CI, confidence interval; ^a^ Range: 0–100; mean (standard deviation): 60.9 (13.4); ^b^ KHEI ≤ 51.6 (the lowest quartile); Model 1: unadjusted model; Model 2: Model 1 + sex + age; Model 3: Model 2 + education + income + marital status + job + working hours + shiftwork + chronic condition + smoking status + physical activity + obesity + perceived stress + survey year.

**Table 4:** The highest and lowest points in the nonlinear analyses (Figure 2) and their coordinates.

	Predicted Values of KHEI		Predicted Probability of Poor Dietary Quality	
	Social Jetlag (X)	Predicted Value (Y) 95% CI	Social Jetlag	Predicted Value (Y) 95% CI
Highest point	18 min	62.6 (62.2, 63.0)	125 min	0.22 (0.20, 0.24)
Lowest point	210 min	61.4 (60.6, 62.2.)	10 min	0.18 (0.17, 0.20)

KHEI, Korean Healthy Eating Index; CI, confidence interval.

## Data Availability

The data are accessible at https://knhanes.kdca.go.kr (accessed on 12 December 2023).

## Supplementary Materials

The following supporting information can be downloaded at: https://www.mdpi.com/article/10.3390/nu16234091/s1, Figure S1. Distribution of the Korean Health Eating Index in the study sample; Figure S2. Q-Q plot of the distribution of the Korean Health Eating Index in the study sample.
