# Supplementary material for: Association of Social Jetlag with the Dietary Quality Among Korean Workers: Findings from a Nationwide Survey
Source: Nutrients. 2024 Nov 27;16(23):4091. doi: 10.3390/nu16234091 (PMC11644551; doi:10.3390/nu16234091)
Supplement: Supplementary file 1 [file nutrients-16-04091-s001.zip › nutrients-3331898-supplementary.pdf]

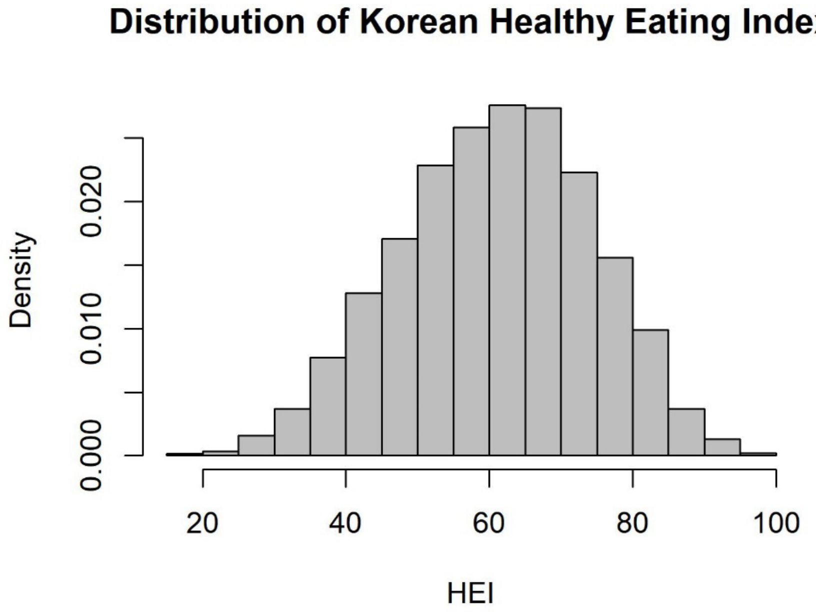

Figure S1 Distribution of the Korean Health Eating Index in the study sample.

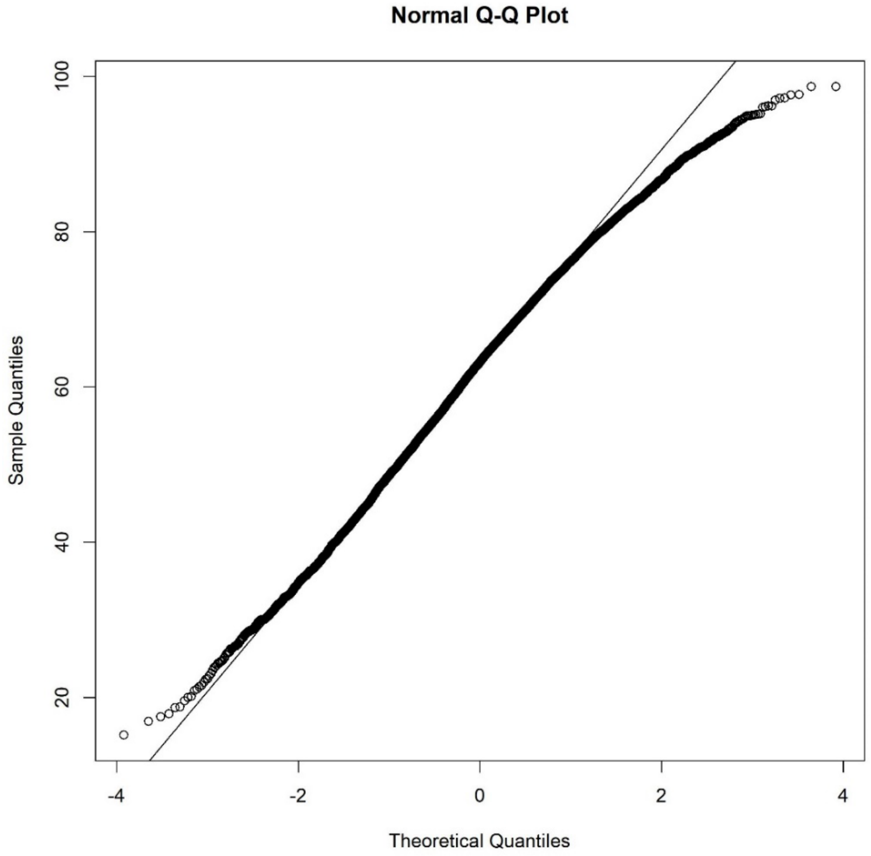

Figure S2 Q-Q plot of the distribution of the Korean Health Eating Index in the study sample.
